# Viromic Insights into Gut RNA Virus Diversity Among Three Corvid Species

**DOI:** 10.3390/v17111508

**Published:** 2025-11-17

**Authors:** Yonggang Dong, Sitong Fan, Lilin Zhu, Kirill Sharshov, Wen Wang

**Affiliations:** 1 State Key Laboratory of Plateau Ecology and Agriculture, Qinghai University, Xining 810016, China; dyg0516@126.com; 2 College of Eco-Environmental Engineering, Qinghai University, Xining 810016, China; fansitong2006@163.com; 3 Xining Wildlife Park of Qinghai Province, Xining 810016, China; zll2025qh@163.com; 4 Federal Research Center of Fundamental and Translational Medicine, Novosibirsk 630117, Russia; sharshov@yandex.ru; 5 Novosibirsk State University, Novosibirsk 630117, Russia

**Keywords:** crow, virus discovery, viral metagenomics, intestinal RNA viruses

## Abstract

As viromics advances, the diversity and ecological significance of RNA viruses in global ecosystems are gaining growing recognition. Nevertheless, studies on RNA viruses in wildlife, especially non-model avian species, are still relatively scarce. This study employed viral metagenomics to systematically characterize the gut RNA viromes of three widely distributed corvid species on the Qinghai–Tibet Plateau: the Red-billed chough (*Pyrrhocorax pyrrhocorax*), Daurian jackdaw (*Coloeus dauuricus*), and Rook (*Corvus frugilegus*). These three corvid species are closely associated with human-inhabited areas on the Qinghai–Tibet Plateau and display distinctive scavenging behaviors that may lower their exposure to environmental pathogens while concurrently elevating their risk of viral infection, rendering them key targets for viral surveillance and research into zoonotic disease transmission. The analysis annotated viral communities into 4 phyla and 8 classes, with *Pisuviricota* and *Kitrinoviricota* emerging as the predominant phyla in all samples. Alpha diversity analysis indicated no significant differences among groups, while beta diversity showed significant compositional differences. KEGG annotation revealed that enriched functional pathways were mainly concentrated in “Global and overview maps”, “Drug resistance: antimicrobial”, and “Biosynthesis of other secondary metabolites”. Furthermore, 4 antibiotic resistance genes and 13 putative virulence factor genes were identified. Phylogenetic analysis further indicated that several identified viruses have the potential for cross-species transmission, underscoring the pivotal role of wild birds in viral ecosystems and disease spread. This study uncovered multi-faceted features of the gut RNA viromes in the three crow species, spanning structural, functional, and evolutionary dimensions. These results offer novel perspectives on the viromes of wild corvids and their potential contributions to viral emergence and dissemination in the Qinghai–Tibet Plateau ecosystem.

## 1. Introduction

Wild birds serve as critical reservoirs for a wide array of pathogens with the potential to cross species barriers, infecting both poultry and humans and presenting a major public health challenge [1]. Among terrestrial animals, birds are unique in their ability to rapidly traverse national and intercontinental boundaries. As they migrate through diverse ecological zones, they can facilitate the spread of various pathogens [2,3], such as viruses [4] (e.g., high pathogenicity avian influenza viruses, West Nile viruses, Newcastle disease viruses), bacteria [5,6] (e.g., *Chlamydia* spp., *Corynebacterium* spp.), and protozoa [7] (e.g., *Toxoplasma gondii*), to new geographic regions, contributing to the wide distribution of these disease agents. Although much research has focused on pathogenic bacteria in wild birds, the viral component of their microbiota—the virome—remains poorly understood, especially when compared to the more extensively studied bacterial microbiome [8]. The virome refers to the entire viral community associated with a host organism, including eukaryotic viruses that infect host cells, bacteriophages that target microbial members of the microbiota, and endogenous viral elements integrated into the host genome [9].

Research on wild bird viruses has predominantly targeted zoonotic or economically impactful pathogens like highly pathogenic avian influenza virus (HPAIV), West Nile virus (WNV), and Newcastle disease virus (NDV), which are associated with major disease outbreaks, cross-species transmission, and global trade disruptions. Avian influenza viruses, for instance, have been endemic in Asia since 2003. One notable event occurred in 2005 at Qinghai Lake, where over 5000 bar-headed geese were reported to have died due to infection with the H5N1 subtype [10]. Given their role as the primary natural reservoirs harboring 17 out of 19 HA (H1–H16, H19) and 9 out of 11 NA (N1–N9) subtypes of avian influenza virus, often without inducing clinical disease, wild waterfowl (Anseriformes, Charadriiformes) are of particular interest in AIV studies [11]. Elmberg et al. conducted a comprehensive review from a One Health perspective on the potential for disease transmission from wild geese and swans to livestock, poultry, and humans, emphasizing that these waterbirds may carry and potentially spread certain pathogens [12].

However, many non-pathogenic or mild pathogenic viruses in other species of wild birds have been largely overlooked in virological research. For example, the virome of wild passerine populations, representing nearly 60% of global avian species, remains largely unexplored, despite their ecological abundance and potential role in viral transmission [13]. Accumulating evidence suggests that many viruses coexist harmlessly or even beneficially with their hosts, influencing immune development, microbiome stability, and overall host health [14]. Understanding the full spectrum of bird viromes is therefore essential not only for identifying emerging threats under the One Health framework [15], but also for discovering the broader ecological and evolutionary roles of viruses in wildlife systems.

Traditional methods for studying virus have long been constrained by several limitations, such as the inability to culture many viruses in vitro, the difficulty in detecting novel or highly divergent viral sequences using conventional PCR techniques, and the lack of broadly reactive antibodies for known viral families [16]. These constraints have significantly hindered comprehensive investigations into viral diversity. In recent years, however, the advent and rapid advancement of viral metagenomic approaches have opened new avenues for unbiased and comprehensive virus detection [17]. Metagenomics allows for the identification of both known and previously unknown viral agents in a wide range of biological and environmental samples, without requiring prior knowledge of the viral genome. This method has been particularly useful for non-invasive investigations using animal-derived samples, including wildlife fecal materials [18].

As the number of published studies highlighting its utility continues to grow, viral metagenomics is increasingly recognized as a powerful tool in virological research. Notably, estimates suggest that approximately 1.67 million viruses from key zoonotic families remain undiscovered among mammalian and avian hosts, with 631,000 to 827,000 of these potentially capable of infecting humans [19]. This staggering number underscores the urgent need for more effective and scalable tools to explore the global virosphere and understand the potential threats posed by emerging viral pathogens. Moreover, advances in viral metagenomic sequencing technologies have enabled researchers to move beyond mere cataloging of viral communities, opening new avenues for exploring the dynamics and functional roles of the virome. Growing evidence indicates that factors such as age, diet, medication use, and co-infections can significantly influence virome composition and activity [20]. As a result, studies focusing on the temporal and environmental variability of viral communities are rapidly expanding, highlighting the importance of understanding the virome as a dynamic and responsive component of the host-microbe ecosystem [21].

Advances in high-throughput sequencing technologies, particularly viral metagenomics, have enabled researchers to transcend the constraints of conventional approaches, allowing systematic characterization of the complex viral communities harbored by wildlife without requiring viral cultivation or prior assumptions, thereby providing novel insights into the field. The Corvidae, a globally distributed family of passerine birds comprising approximately 131 species, are renowned for their exceptional adaptability and intelligence [22]. Their omnivory and facultative scavenging behavior confer exceptional dietary flexibility, ranging from natural sources such as insects, fruits, and carrion to anthropogenic food waste in urban environments, which significantly contributes to their success in human-altered landscapes [23]. Their advanced cognitive abilities, including observational learning, further enhance their capacity to exploit urban resources and thrive in close proximity to humans [24]. Consequently, many corvid species are classified as urban adaptors or even exploiters, making them key subjects for studying the ecological and evolutionary impacts of urbanization [25]. Owing precisely to their distinctive ecological and behavioral characteristics, they also constitute ideal models for investigating viral transmission and evolution.

Notably, the Red-billed chough, Daurian jackdaw, and Rook are widely distributed throughout the Qinghai–Tibet Plateau, occupying habitats that overlap extensively with human settlements; they display high mobility, strong social tendencies, and commonly scavenge on livestock carcasses and domestic refuse [26]. Such facultative scavenging behavior enhances their likelihood of encountering pathogens. Moreover, existing research indicates that the Rook’s frequent foraging at landfill sites heightens its exposure to polluted environments, thereby increasing its propensity to harbor multiple pathogens, including *Escherichia coli* (*E. coli*) [27].

While they provide important ecosystem services such as seed dispersal and environmental monitoring, they may also contribute to ecosystem disservices, including noise pollution, waste scavenging, and, notably, disease transmission. For example, multiple *Sarcocystis* species infections are prevalent among corvids, suggesting that these birds could be significant vectors in the transmission dynamics of such parasites [28]. In a separate study, Chang et al. utilized metatranscriptomic techniques to detect potential viral pathogens linked to corvid populations [29]. Our research group has previously conducted a comparative study of the gut microbiome among five common crow species on the Qinghai–Tibetan Plateau using metagenomic approaches. Thess studies revealed the presence of numerous clinically relevant pathogens [6] and antibiotic resistance genes [26], highlighting significant public health concerns. The adaptability of Corvidae birds to human-dominated environments, coupled with their potential role in disease transmission, not only makes them crucial subjects from a conservation perspective but also positions them as key players in public health dynamics. In contrast to bacteria and parasites, viruses possess higher mutation rates, a greater capacity for cross-species transmission, and frequently establish long-term asymptomatic infections in their hosts—features that render them challenging to detect with traditional diagnostic approaches. Consequently, a systematic characterization of their virome is crucial for evaluating zoonotic spillover risk and advancing our understanding of viral ecology.

However, there exist gaps in the knowledge of virus diversity in these bird species in this region. To address this knowledge gap regarding the gut virome of wild Corvidae species, we conducted a comprehensive RNA viromic investigation of three widely distributed birds on the Qinghai–Tibetan Plateau: the Red-billed chough, Daurian jackdaw, and Rook. In this study, we analyzed samples collected from three corvid species to: (i) characterize their RNA viromes, (ii) to comparatively assess viral diversity and abundance across species, and (iii) evaluate the influence of host taxonomy on virome structure, identify putative animal-origin or zoonotic viruses, and assess their potential for emergence and transmission within the alpine ecosystem. By integrating metagenomic sequencing with ecological analyses, this work provides new insights into the viral communities harbored by wild corvids and their potential role in viral emergence and transmission in Qinghai–Tibetan Plateau ecosystems.

## 2. Materials and Methods

### 2.1. Sample Collection

**Figure 1:**
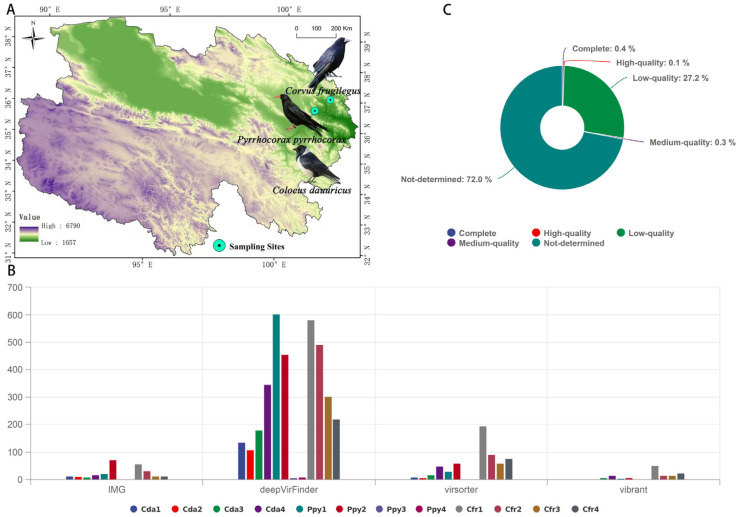
Sampling and sequence assessment. (**A**) Sampling distribution of the three corvid species. (**B**) Number of viral candidate sequences identified by four categories of tools. (**C**) Circos plot showing the completeness of viral contigs as assessed by CheckV.

In a specific region of Qinghai Province, China, we captured a total of 12 wild corvid individuals, with four individuals (*n* = 4) from each species: Rook, hereafter abbreviated as Cfr; Red-billed chough, abbreviated as Ppy; and Daurian jackdaw, abbreviated as Cda (Figure 1A). All sampled individuals were adults drawn from their native wild populations. Sampling locations were geographically distinct: Rooks (Cfr, *n* = 4) were collected in Huzhu County, Qinghai Province, China (101°54′10″ E, 37°4′10″ N); Red-billed choughs (Ppy, *n* = 4) in Huangyuan County, Qinghai Province, China (101°16′43″ E, 36°40′1″ N); and Daurian jackdaws (Cda, *n* = 4) at a separate site also within Huangyuan County, Qinghai Province, China (101°17′26″ E, 36°41′21″ N). At the time of capture, all individuals were observed to be in good health. Following capture, individuals were humanely euthanized via cervical dislocation, and subsequent dissection was carried out under sterile conditions to aseptically remove the entire intestinal tract and carefully collect the luminal contents. All procedures were conducted while wearing masks and disposable gloves to minimize the risk of cross-contamination. Upon collection, samples were immediately transferred to an ultra-low temperature freezer at −80 °C for long-term storage prior to downstream experimental analyses.

### 2.2. Viral RNA Extraction and Sequencing

Viral particles in the collected fecal samples were purified and concentrated following a previously established protocol [30,31] to eliminate potential contaminants, including host cells, bacterial components, and undigested food residues. The procedure was as follows: fecal samples were resuspended in sterile phosphate-buffered saline (PBS) at a 1:5 (*v*/*v*) ratio, thoroughly homogenized to form a suspension, and underwent three repeated freeze–thaw cycles to promote the release of viral particles. The suspension was then centrifuged at 12,000× *g* for 5 min at 4 °C to pellet cellular debris and large particulate contaminants. The supernatant was collected and sequentially filtered through 0.45 μm and 0.22 μm pore-size filters to remove residual microbial cells and particulates. The filtrate was transferred to an ultracentrifuge tube containing a 28% (*w*/*w*) sucrose cushion and subjected to ultracentrifugation at 160,000× *g* for 2 h at 4 °C in a HIMAC CP 100WX ultracentrifuge (Hitachi, Tokyo, Japan) to pellet and concentrate viral particles. Following centrifugation, the supernatant was carefully removed, and the pellet was resuspended in 200 μL of stabilization buffer (SB buffer). An appropriate volume of enzyme mix buffer (EMB) and enzyme mix (EM) was then added, and the suspension was incubated at 37 °C for 1 h to selectively degrade non-viral nucleic acids. The reaction was terminated by adding 2 μL of stop solution (SS), followed by thorough mixing and incubation at 65–75 °C for 10 min to inactivate the enzymes. The sample was then centrifuged at 2000× *g* for 5 min, and 200 μL of the resulting supernatant was transferred and stored at −20 °C for downstream analysis. Viral RNA was extracted using the TaKaRa MiniBEST Viral RNA Extraction Kit Ver.5.0, (TaKaRa Bio Inc., Kusatsu, Japan) and an estimated input of approximately 4 ng of total RNA was used for whole transcriptome amplification with the Qiagen REPLI-g Cell WGA & WTA Kit. (QIAGEN GmbH, Hilden, Germany) The quality and concentration of the amplified products were measured using a NanoDrop spectrophotometer (Thermo Fisher Scientific, Waltham, MA, USA), and their integrity was further verified by 1.5% agarose gel electrophoresis. Sequencing libraries were constructed from qualified samples following the manufacturer’s instructions of the ALFA-SEQ DNA Library Construction Kit (Ark Biosciences, Knoxville, TN, USA). All libraries were sequenced on the Illumina NovaSeq 6000 platform, with library construction and sequencing conducted by Megene Biotechnology Co., Ltd. (Guangzhou, China).

### 2.3. Bioinformatics and Statistical Analysis

#### 2.3.1. Raw Data Processing

Paired-end raw sequencing reads were subjected to quality filtering using Trimmomatic (v0.36) [32] with the parameters: ILLUMINACLIP:adapters.fa:2:30:10 SLIDINGWINDOW:4:15 MINLEN:75. Filtered high-quality reads were mapped to the Large-billed Crow reference genome (DRR250114) using BWA (v0.7.12-r1039) [33] to eliminate host-derived sequences. De novo assembly and identification of viral sequences. De novo assembly of host-depleted reads was conducted using Megahit (v1.1.1) [34], with a minimum contig length threshold of 500 bp. Candidate viral contigs were identified from assembled sequences exceeding 1000 bp in length using a combination of tools: DeepVirfinder (score > 0.7 & *p*-value < 0.05) [35], VirSorter2 (v2.0) [36], VIBRANT (v1.2.1) [37], and IMG/VR (v4.0) (using Blastn alignment). DeepVirFinder (v1.1) is a deep learning—based tool whose training dataset includes 2314 prokaryotic virus genomes from the NCBI RefSeq database (released before January 2014), supplemented with additional sequences deposited between January 2014 and May 2015, as well as approximately 1.3 million viral contigs assembled from metaviromic studies [38]. Candidate viral sequences were clustered into vOTUs using Phageannotator (v1.4) with thresholds of 95% sequence identity and 85% coverage, and the longest contig within each cluster was designated as the representative sequence [39]. The completeness and novelty of each vOTU were evaluated using CheckV (v0.7.0) to determine its placement within established viral lineages [40].

#### 2.3.2. Viral Abundance Quantification and Functional Annotation

Cleaned reads were aligned to vOTUs using Bowtie2 (v2.3.4.3) [41], and abundance was quantified using SAMtools (v1.9) [42]. Viral relative abundance was reported as TPM (transcripts per million mapped reads) [43]. The taxonomic classification of vOTUs was determined using geNomad (v1.5.1) [44]. Gene prediction was carried out using metaProdigal (v2.6.3) under default settings [45]. Functional annotation was performed as follows: protein domains were identified using PfamScan (v1.6) (e-value < 1 × 10^−5^, score > 40) based on HMM models; KO numbers were assigned using KEGG Ghost-KOALA (v3.1); and gene sequences were aligned against the CAZy, SARG, and VFDB databases using DIAMOND (v0.9.22.123) [46] to annotate carbohydrate-active enzymes, antibiotic resistance genes, and virulence factors, respectively.

#### 2.3.3. Viral Community Diversity and Phylogenetic Analysis

Alpha-diversity indices (e.g., species richness, Shannon index) were computed at the vOTU level using the vegan package (v2.5.7) to evaluate viral diversity within individual samples. Beta-diversity was analyzed using Bray–Curtis dissimilarity to visualize viral community structural differences across samples, and the significance of group-level differences was evaluated using PERMANOVA (v0.2.0) via the adonis function in the vegan package (1000 permutations). For phylogenetic analysis, multiple sequence alignment was conducted using MAFFT (v7.475), and reference sequences were selected using ViPTree (v4.0) [47], a tool that computes whole-genome similarity based on tBLASTx (v2.13) alignment results. Multiple sequence alignment was carried out using MAFFT (v7.475) under default settings. Phylogenetic trees were inferred using IQ-TREE (v2.3.4) [48], with the ModelFinder Plus (MFP) algorithm employed to automatically determine the optimal substitution model; branch support was assessed using the ultrafast bootstrap method with 1000 replicates.

## 3. Results

### 3.1. Subsection Overview of Virome Sequencing Data

In this study, twelve sequencing libraries (each corresponding to one intestinal sample) yielded a total of 878,961,070 raw reads. After quality control and removal of host-derived sequences, 148,787,388 clean reads were obtained. On average, 36.08% of host sequences were removed in the Ppy group, 32.22% in the Cda group, and 65.38% in the Cfr group (Table S1). A total of 4412 contigs exhibiting viral features were identified using four distinct methods. The Cfr group harbored the highest number of viral contigs, followed by the Ppy group, while the Cda group had the lowest. deepVirFinder detected the highest number of viral contigs among the three groups (Figure 1B). Quality assessment of these viral sequences using CheckV revealed that 0.1% were high-quality genomes, 0.3% were medium-quality, and 27.2% were low-quality sequences (Figure 1C). After excluding sequences of “not-determined” quality and performing redundancy reduction, a total of 613 non-redundant viral operational taxonomic units (vOTUs) were obtained, with an average length of 938.8 bp, an N50 of 935 bp, and an average GC content of 43.82%.

### 3.2. Composition of Viral Community Structure

**Figure 2:**
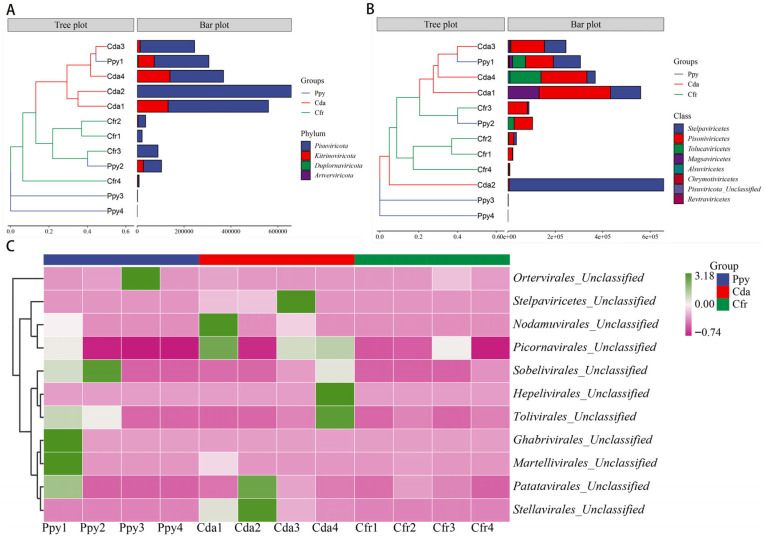
Taxonomic annotation of RNA viromes in the three corvid species at multiple classification levels. (**A**) Composition of viral communities at the phylum level. (**B**) Composition of viral communities at the class level. (**C**) Composition of viral communities at the unclassified family level.

**Figure 3:**
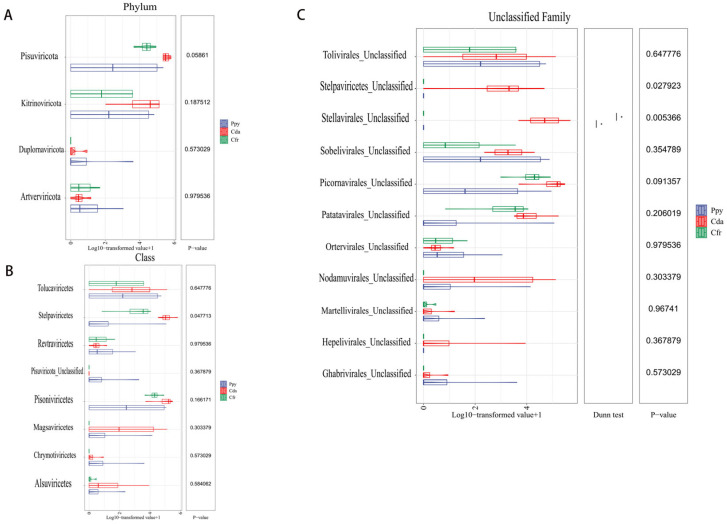
Abundance difference tests of RNA viral communities among the three corvid groups. (**A**) Difference test at the phylum level. (**B**) Difference test at the class level. (**C**) Difference test at the unclassified family level. Statistical method: Kruskal–Wallis test, with *p*-values adjusted by the Benjamini–Hochberg (BH) procedure. * *p* < 0.5.

Taxonomic annotation showed that the viral communities across the three crow species were classified into 4 phyla and 8 classes. At the phylum level, *Pisuviricota* (83.6%) was the most abundant, followed by *Kitrinoviricota* (16.17%), *Duplornaviricota* (0.17%), and *Artverviricota* (0.05%). Within each group, *Pisuviricota* and *Kitrinoviricota* were the predominant phyla, especially in the Cfr and Cda groups where *Pisuviricota* dominated (Figure 2A). At the class level, the relative abundances were ranked as follows: *Stelpaviricetes* (43.35%), *Pisoniviricetes* (40.17%), *Tolucaviricetes* (9.35%), and *Magsaviricetes* (6.45%). The remaining four classes (*Chrymotiviricetes*, *Alsuviricetes*, *Revtraviricetes*, *Pisuviricota_Unclassified*) had abundances of less than 1%. Across the three groups, *Pisoniviricetes* and *Stelpaviricetes* were the dominant classes within the viral communities (Figure 2B). Sequences were classified at the family level as *Solemoviridae* and *Potyviridae*. Furthermore, a considerable fraction of viral sequences remained unclassified at the family level and were instead annotated as unclassified taxa within distinct orders, predominantly *Picornavirales_Unclassified* (34.55%), *Stellavirales_Unclassified* (26.24%), and *Patatavirales_Unclassified* (13.42%) (Figure 2C). To assess intergroup abundance differences, Kruskal–Wallis tests were conducted at the phylum and class levels, with p-values corrected using the Benjamini–Hochberg method. No significant differences were observed among the groups at the phylum (Figure 3A) or class levels (Figure 3B). However, within the unclassified families, a significant difference was detected for *Stellavirales_Unclassified* (*p* = 0.005) (Figure 3C).

**Figure 4:**
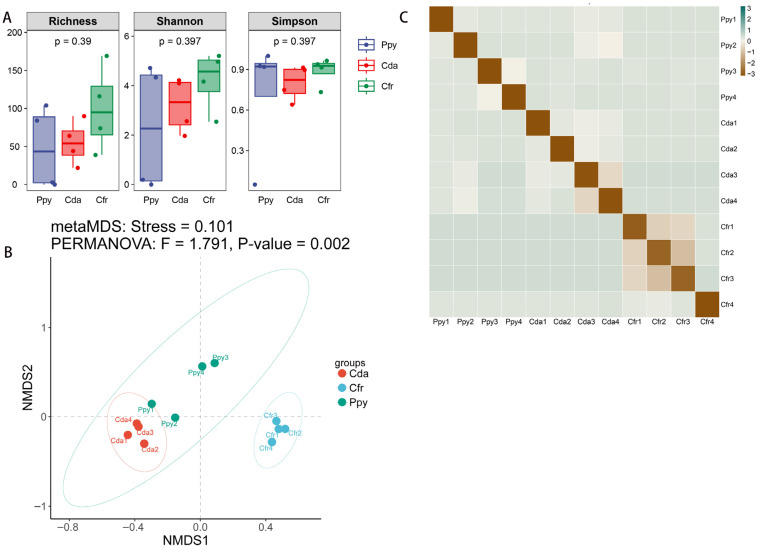
Viral community diversity analysis. (**A**) Boxplots showing α-diversity of viral communities in the two corvid species based on Richness, Shannon, and Simpson indices. (**B**) Non-metric multidimensional scaling (NMDS) analysis based on Bray–Curtis dissimilarity. (**C**) Heatmap of Bray–Curtis distances among all samples.

To compare the overall diversity of viral communities across the three crow groups, we computed diversity indices at the vOTU level. The Richness index quantifies the number of distinct viral sequences per sample, indicating community richness; the Shannon index reflects both diversity and evenness; and the Simpson index measures the dominance and uniformity of abundant sequences, collectively characterizing viral community diversity. The Cfr group exhibited higher α-diversity (Richness: 99.5 ± 56.014, Shannon: 4.221 ± 1.203, Simpson: 0.887 ± 0.105) compared to the Cda (55 ± 28.959, 3.212 ± 1.123, 0.8 ± 0.129) and Ppy groups (47.75 ± 54.039, 2.31 ± 2.563, 0.723 ± 0.451), indicating greater viral richness, evenness, and overall diversity. The Ppy group not only showed the lowest diversity but also the highest variability, suggesting a less stable community structure. Intergroup comparison tests revealed no statistically significant differences among the three groups for any of the three α-diversity indices (*p* > 0.05) (Figure 4A). Non-metric multidimensional scaling (NMDS) analysis based on Bray–Curtis dissimilarity revealed significant differences among the three groups (*p* = 0.002). The samples showed clear spatial separation, indicating distinct viral community structures. The Cfr group was most distinct from the other two, while the Ppy and Cda groups partially overlapped but were still separable (Figure 4B,C).

### 3.3. Functional Overview of the Virome

**Figure 5:**
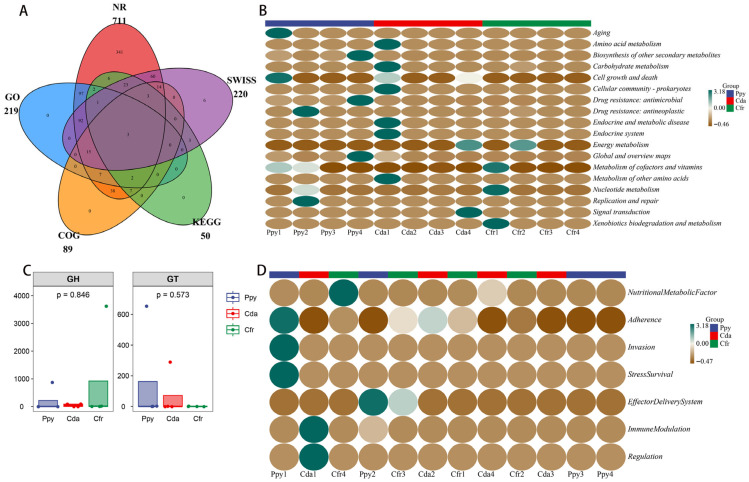
Gene prediction and functional annotation. (**A**) Venn diagram showing the number of annotated genes identified across five databases used for virome annotation. (**B**) Clustered heatmap of KEGG metabolic pathways at level 2. (**C**) Boxplots comparing the abundance of two types of carbohydrate-active enzymes (CAZymes) detected between the two groups. (**D**) Heatmap showing the modular functional distribution of virulence factor genes (VFGs) across all samples.

Gene prediction was conducted on RNA viral genomes from three corvid species, resulting in the identification of 1017 coding sequences (CDSs). These CDSs totaled 519,570 bp in length, with an average length of approximately 510 bp. The viral genomes displayed high gene density, with an average of 1.767 genes per kilobase. Predicted CDS regions covered 90.3% of the total assembled sequence length, amounting to 55,916 bp. Furthermore, the average GC content of the CDSs was 43.9%. Functional annotation was performed by aligning the 1017 unigenes against five public databases: NR, Swiss-Prot, KEGG, COG, and GO. Results indicated that 720 unigenes (70.8%) were assigned functional annotations in at least one database. The NR database provided the highest annotation rate (69.91%), whereas KEGG and COG had lower rates (4.92% and 8.75%, respectively), suggesting limited resolution in metabolic pathway and orthologous group assignment, with nearly 30% of genes lacking functional annotation (Figure 5A).

KEGG annotation revealed that the most abundant viral gene functions were enriched in “Global and overview maps” (33.06%), “Drug resistance: antimicrobial” (32.35%), and “Biosynthesis of other secondary metabolites” (30.02%); all other functional pathways had abundances under 1%. Across all three groups, “Global and overview maps” was the most dominant functional category (Figure 5B). This indicates that the functional profiles of RNA viral genes in the three crow groups are highly concentrated in these three pathways, suggesting an active role for viruses within the host intestinal microenvironment. SARG annotation identified four antibiotic resistance genes (ARGs) with assigned functions (Table S2). β-lactam resistance genes were the main feature; in the Ppy group, fully matching extended-spectrum β-lactamase genes TEM-162 and TEM-191 were detected, with high confidence (E-value < 1 × 10^−75^), suggesting their stable presence in the samples. Additionally, the ugd gene was identified in the Cda group. A mutated gene homologous to the DNA gyrase GyrB subunit (parY) was also detected in the Ppy group. These findings suggest that the viral communities in these three groups harbor diverse resistance determinants and have the potential for multidrug resistance.

Analysis based on the CAZy database identified one glycoside hydrolase (GH) and one glycosyltransferase (GT) encoded by viral genes. No significant intergroup differences were observed for these enzymes (Figure 5C). Annotation using the VFDB database identified 13 putative virulence factor genes, categorized into 7 functional types. Key virulence traits included Type VI/VII secretion systems, LPS modification genes, and adhesins. The results revealed a diverse repertoire of virulence factors (Figure 5D), spanning critical pathogenic steps including adhesion, invasion, immune evasion, effector delivery systems, and stress survival. Nutritional Metabolic Factors were the most abundant category (61.62%), followed by Immune Modulation (14.77%).

### 3.4. Evolutionary Analysis

**Figure 6:**
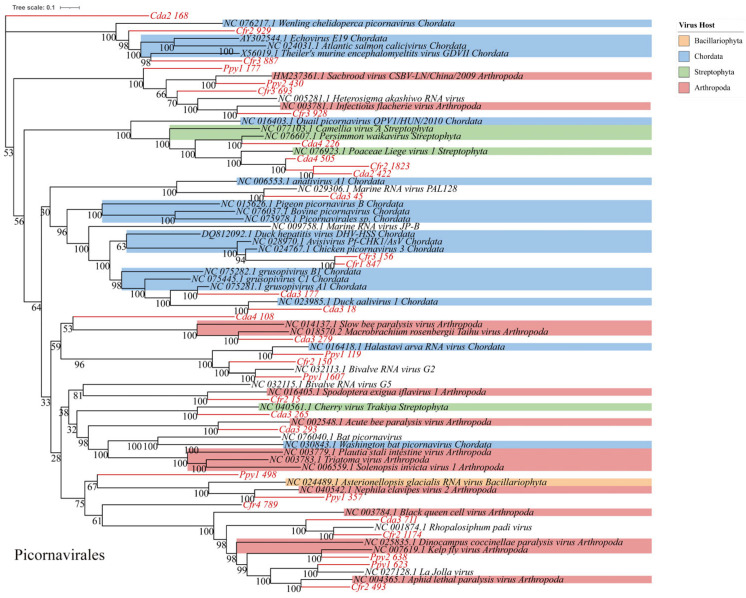
Phylogenetic analysis. Maximum-likelihood phylogenetic tree of *Picornavirales*. Viral operational taxonomic units (vOTUs) identified in this study are highlighted in red. Colored blocks indicate the known host taxa of reference sequences only (e.g., Arthropoda, Chordata, Streptophyta), as documented in public databases. Bootstrap support values (based on 1000 replicates) are shown at major nodes.

**Figure 7:**
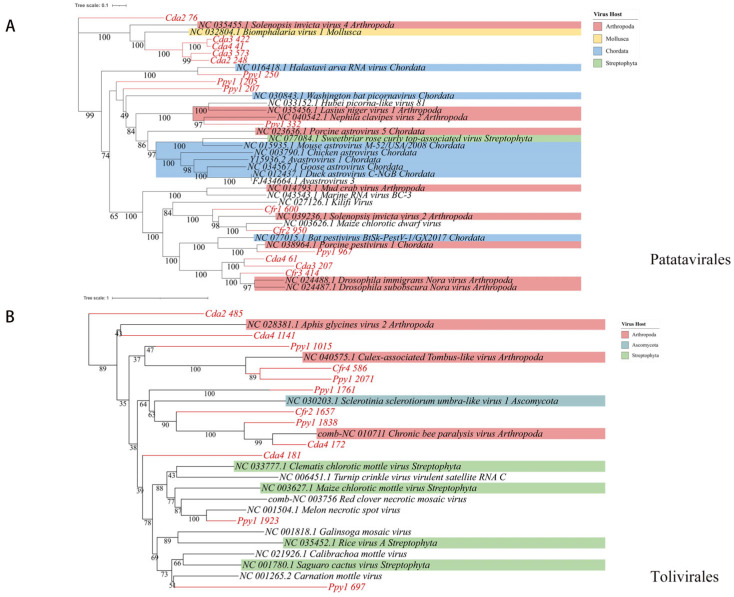
Phylogenetic analysis. Maximum-likelihood phylogenetic trees of (**A**) *Patatavirales* and (**B**) *Tolivirales*.

RNA viruses within the phylum *Pisuviricota* generally encode homologous RNA-dependent RNA polymerases (RdRp) that belong to the Pfam RdRp_1 family. The majority of these viruses possess icosahedral capsids with T = 1 to T = 4 symmetry. Further analysis revealed 69 representative viral operational taxonomic units (vOTUs), classified into four orders: *Picornavirales* (*n* = 32), *Patatavirales* (*n* = 15), *Sobelivirales* (*n* = 14), and *Stellavirales* (*n* = 5). Among these, *Picornavirales* is an order encompassing numerous significant viruses. Phylogenetic analysis indicated that these sequences display notable genetic distances from viral sequences previously identified in various host groups. Within these host groups, arthropods (blue) and chordates (pink) constitute a significant proportion, indicating that many *Picornavirales* strains can infect both arthropods and chordates. The presence in other host lineages highlights the broad host range of these viruses (Figure 6), implying a potential risk for cross-species transmission. Phylogenetic analysis of *Patatavirales* revealed that the identified sequences are closely related (Figure 7A). Single-stranded RNA viruses within the phylum *Kitrinoviricota* are characterized by homologous RNA-dependent RNA polymerases (RdRPs) from the Pfam RdRP_2 or RdRP_3 families. Their capsid architectures comprise both icosahedral (with T = 1, T = 3, or T = 4 symmetry) and helical forms. A total of 18 representative viral operational taxonomic units (vOTUs) were identified, assigned to four orders: *Tolivirales* (*n* = 12), *Nodamuvirales* (*n* = 4), *Martellivirales* (*n* = 1), and *Hepelivirales* (*n* = 1). Phylogenetic analysis of *Tolivirales* revealed that these sequences display varying levels of genetic divergence from viruses found in hosts across Arthropoda, Streptophyta, and Ascomycota (Figure 7B), indicating a potentially wide host range or environmental prevalence.

## 4. Discussion

With the development of viral metagenomics, the diversity and ecological significance of RNA viruses in global ecosystems have received growing attention [17]. Although prior studies have revealed the widespread distribution of RNA viruses in environments such as oceans, soils, and the human gut, investigations into RNA viruses in wildlife, particularly non-model birds, have remained relatively limited. This study conducted a metagenomic analysis of the gut RNA viromes from three corvid species, systematically characterizing the composition, functional potential, and evolutionary features of their viral communities. Regarding viral community composition, *Pisuviricota* and *Kitrinoviricota* represented the dominant viral phyla in all samples. These two phyla have been previously detected in diverse biological groups, including marine organisms [49,50], mammals [51], arthropods [52] and environmental types [53]. A study of the viromes from 56 birds and 91 small mammal species on the Tibetan Plateau demonstrated that viral families within *Pisuviricota* and *Kitrinoviricota* (e.g., *Picornaviridae*, *Picobirnaviridae*) were highly abundant and represented a major component of the RNA viral communities [54]. In comparison with our results, this suggests that these two viral phyla exhibit broad host adaptability and wide ecological distribution, potentially fulfilling key ecological functions across diverse ecosystems.

Notably, among the annotated gut viral communities of the three crow species, *Potyviridae* and *Solemoviridae* were classified at the family level, and both are of plant origin. *Potyviridae* is an RNA virus family that infects a wide range of plants; its virions are flexuous filaments approximately 650–950 nm long and 11–20 nm in diameter, exhibiting helical symmetry. The family comprises over 10 genera and 230 species and is transmitted by various vectors, including aphids, whiteflies, mites, and plasmodiophorids [55]. *Solemoviridae* virions are approximately 26–34 nm in diameter, with capsids formed by 180 capsid protein subunits arranged in T = 3 symmetry, and this family infects a broad range of crops and wild plants [56]. Recently, novel *Solemoviridae*-related viruses have been identified in insects, which exhibit phylogenetic associations with plant *Solemoviridae* viruses in their RNA-dependent RNA polymerase (RdRp) sequences, indicating a possible insect-plant transmission pathway [57]. Corvids are omnivorous birds that commonly feed on plant fruits, seeds, and crop residues. Given this foraging behavior, we detected RNA fragments belonging to *Potyviridae* and *Solemoviridae* in their gut contents, suggesting dietary exposure resulting from the consumption of virus-infected plant material. Nevertheless, while the presence of plant viruses is tentatively ascribed to dietary sources, potential contributions from environmental exposure or other non-infectious pathways cannot be excluded. Prior research has not established a link between diet and virome composition in wild birds, although a study on bat viromes and dietary habits revealed a strong correlation between viral community composition and diet [58]. The influence of diet on host viral community diversity is a significant factor in avian virome research and should not be overlooked, particularly in omnivorous species. Unfortunately, our study focused solely on viral community characterization and did not collect detailed dietary data during sampling; future work could utilize amplicon sequencing to explore the dietary composition of corvids and its relationship with their viromes.

Moreover, the sequences assigned to these two families represent putative *Potyviridae* and *Solemoviridae* members, with their RdRp-encoding regions showing genetic divergence from known viruses. Sequence Cda2__248 shared only 64.94% amino acid identity with its closest known relative, whereas Ppy1__1325 showed even lower similarity (31.09%), with most matching sequences annotated as hypothetical proteins. Phylogenetic analysis further supports their unique evolutionary placement within their respective families. However, as these sequences originated from single samples and are fragmented contigs lacking conserved structural protein genes, and given the absence of established species demarcation criteria for the RdRp of such poorly characterized viral groups, furthermore, RNA viruses exhibit high sequence diversity, which hinders reliable sequence alignment and classification at low similarity thresholds. Although phylogenetic analysis offers preliminary evidence, given the extremely small number of these annotated sequences, we do not classify them as new viral species [59,60]. Nevertheless, this result indicates the existence of genetically distinct RNA virus lineages within viromes associated with corvids. Future efforts involving broader sampling and complete genome assembly will facilitate the determination of the taxonomic status, host range, and ecological roles of these viruses.

Although viral community annotations in the three corvid species revealed diverse structural compositions, α-diversity analyses showed no significant differences among the three groups for any index (*p* > 0.05), suggesting a relatively consistent overall diversity of gut RNA viromes. The number and distribution of viral types within individual birds were thus broadly similar across species. However, interestingly, NMDS analysis using Bray–Curtis distances clearly demonstrated significant structural differentiation among the three viral communities (*p* = 0.002). The Cfr group formed a distinct cluster, whereas Ppy and Cda, though partially overlapping, were still distinguishable. It has been established that the composition of the gut virome can vary substantially in response to changes in diet, physiological status, and environmental factors [61]. We hypothesize that the structuring of viral communities in these three corvid groups may be influenced not only by host-specific factors but also, more prominently, by extrinsic factors including ecological environment, feeding behavior, and geographic distribution. A study on the fecal viromes of migratory wild ducks found that variations in viral communities were attributable to specific ecological niches and dietary sources [62]; the marked separation of the Cfr group from Ppy and Cda in our NMDS results may similarly reflect such ecological drivers. Cfr group individuals may be influenced by their local habitat or utilize distinct food sources, resulting in exposure to specific viral lineages and consequently shaping a unique gut virome structure. Due to the limitations of our experimental design, we cannot definitively identify the specific factors responsible for the differences in viral communities among the three groups. Future research should employ long-term monitoring studies that incorporate additional ecological data, such as habitat utilization, dietary composition, environmental parameters, and host physiological metrics to elucidate the key factors driving virome divergence in these avian species. This pattern characterized by non-significant intergroup differences in alpha diversity but significant divergence in beta diversity aligns with results previously reported in studies of bacterial communities [63,64]. This trend suggests that the viral communities in these three corvid species may maintain a relatively stable level of overall diversity while remaining responsive to structural shifts induced by host-specific or environmental variables. Furthermore, it is important to note that our relatively small sample size may limit statistical power, meaning the findings from the alpha and beta diversity analyses should be interpreted as preliminary.

Functionally, KEGG annotation showed that the viral communities were enriched in pathways related to “Global and overview maps,” “Drug resistance: antimicrobial,” and “Biosynthesis of other secondary metabolites.” We speculated that the enrichment of these functional categories could be linked to the corvids’ facultative scavenging behavior and their adaptation strategies for surviving in extreme environments like the high-altitude, low-oxygen conditions of the Tibetan Plateau. Earlier research on scavenging birds in Antarctica demonstrated that feeding on carcasses could elevate the risk of exposure to intestinal pathogens in wild birds [65]. When feeding on carcasses, corvids are exposed to a greater risk of pathogen invasion. A previous study on the scavenging behavior of Eurasian magpies reported that Enterococcus faecalis present in the birds can modulate gut microbial composition, subsequently influencing host anti-inflammatory and immune responses, and proposed this bacterium as a potential biomarker linked to scavenging activity [66]. We hypothesized that viruses might contribute to maintaining gut microbial stability by carrying or regulating genes involved in core metabolic processes, acting in concert with gut bacteria under conditions of repeated pathogen exposure. Furthermore, evidence has shown that coronaviruses can manipulate host basal metabolic pathways through protein–protein interactions with factors involved in RNA metabolism, transcriptional control, and translation, thereby enhancing their ability to replicate within cells and evade immune responses [67]. Although direct evidence for widespread viral participation in secondary metabolism is currently lacking, these interactions closely linked to primary metabolism suggest that viruses may adapt to specific environmental pressures by rewiring host metabolic networks. Likewise, the precise mechanisms by which these viral functions affect host physiological adaptation remain unclear; nevertheless, their presence hints at a potential role of the virome in host resilience under environmental stress, warranting further investigation in future research.

Notably, definitive ARGs were identified in the Ppy group samples, including the extended-spectrum β-lactamase genes TEM-162 and TEM-191, along with homologs of ugd and parY. TEM-191, in particular, was previously reported in multidrug-resistant Klebsiella pneumoniae from red kangaroos and resides on a mobile plasmid, indicating possible transmission via animal sources and a potential threat to public health [68]. Given their ability to harbor diverse parasites, viruses, and bacteria, corvids function as significant reservoirs and potential transmission vectors. Considering the substantial overlap between their ecological niches and human settlements, their potential role as vectors for the dissemination of antibiotic resistance genes cannot be ignored. We hypothesize that corvids may represent a long-underestimated hub in the spread of antimicrobial resistance within the “One Health” context linking natural environments and human communities. Regarding the possibility that crows may contribute to the spread of resistance genes, future studies should conduct more systematic surveillance and source-tracking analyses to assess their actual transmission risk. Regrettably, as with earlier findings, the small sample size and restricted study scope mean that the present functional and ecological interpretations are still preliminary and should be treated cautiously. Specifically, the putative role of corvids as transmission vectors for antibiotic resistance genes needs to be confirmed through future multi-omics and cross-regional studies.

The following limitations of this study must be acknowledged. While these three facultative scavenger species on the Qinghai–Tibet Plateau are ecologically important for maintaining balance and reducing pathogen spread, the region’s rugged topography and extreme conditions, combined with the animals’ high alertness, pose significant challenges for field sampling. Additional care required to ensure bird welfare during capture further complicates the process. Although the small sample size may have limited statistical power to some extent. Recent research [69] indicates that well-designed experiments and robust statistical approaches could still detect microbial community differences despite limited sample sizes. Additional studies have confirmed that microbial communities could be effectively characterized with small sample numbers [70,71,72]. Second, metagenomic techniques inherently produce large volumes of data [73], making effective data utilization and analysis crucial. Future research based on larger sample sizes could help overcome some of the current limitations and potentially reveal new insights. At the same time, although we used multiple tools to identify viral candidate sequences and applied stringent software parameters, the potential influence of sample size remained a concern; thus, the conclusions of this study were regarded as preliminary and exploratory. Future studies should employ larger sample sizes to complement and expand upon our findings. It should be emphasized that this study lacked negative (blank) controls, precluding direct assessment of potential contamination from reagents or environmental sources. Nonetheless, several precautions were taken to mitigate potential biases. Sample collection and nucleic acid extraction were carried out under strict aseptic conditions. Viral particles were enriched through sequential centrifugation and filtration steps designed to eliminate host cells, bacteria, and other particulate impurities. Host-derived sequences were subsequently filtered out in silico by mapping reads to available reference genomes. Candidate viral sequences were identified using a suite of complementary computational tools to minimize false-positive assignments. The freeze–thaw–vortexing procedure coupled with nuclease treatment may have selectively depleted structurally fragile viral particles, including enveloped viruses. In the absence of exogenous spike-in controls, viral recovery efficiency could not be accurately quantified. Therefore, interpretations of viral relative abundance should be made with caution.

An additional objective constraint stemmed from the multiple challenges inherent in viral metagenomics, such as high costs, the risk of sample contamination [74], inefficient capture of low-abundance viral communities—particularly in environmental samples where standard nucleic acid extraction protocols may poorly recover target viruses [75] and the scarcity of viral databases, which limited the accuracy of annotation results [76]. In addition, a substantial proportion of viral contigs in this study were assigned an “undetermined” quality status by CheckV. This outcome largely stems from their relatively short lengths and lack of conserved viral marker genes, which hindered CheckV’s ability to reliably infer genome completeness against reference databases. Furthermore, this limitation is intrinsic to CheckV: its reference database is heavily skewed toward known viruses, making it poorly suited for accurately evaluating highly divergent or putatively novel viral sequences [40]. Notwithstanding this constraint, we chose to employ CheckV for quality assessment, as it remains the most widely adopted and comparatively reliable tool in metagenomic research for estimating viral genome completeness and detecting host-derived contamination. It is important to note that the distribution of quality classifications could vary if alternative tools—such as VirSorter2, VIBRANT—were employed, owing to differences in their underlying algorithms and evaluation metrics. Although rigorous experimental design and analytical workflows were employed to mitigate technical limitations as much as possible, the inherent challenges of viral metagenomics may still have impacted the comprehensiveness and precision of the findings. Nonetheless, this study offered valuable preliminary insights into the viral community structure, potential virus–host interactions, and inferred ecological functions of these three corvid species, establishing baseline data for understanding the gut RNA viromes of facultative scavenging birds.

## 5. Conclusions

Overall, this study uncovered multi-faceted features of the gut RNA viromes in three crow species, encompassing structural, functional, and evolutionary dimensions. The virome structure uncovered both shared and distinct features across the three crow species. The detection of antibiotic resistance genes and virulence factors indicates potential roles in modulating microbial communities and posing public health concerns. Although no viral sequences exhibiting unambiguous zoonotic potential were detected, the possible role of wild birds in viral transmission networks merits further exploration, considering their frequent interactions with diverse ecological niches. Our findings offer novel perspectives on the viral communities harbored by wild corvids and their potential contributions to viral emergence and dissemination within the Qinghai–Tibet Plateau ecosystem. Future research should examine the potential role of these avian species—especially those in close proximity to human habitation—in viral transmission networks, including their possible participation in zoonotic transmission cycles.

## Data Availability

The raw sequence data reported in this paper have been deposited in the Genome Sequence Archive in National Genomics Data Center, China National Center for Bio-information/Beijing Institute of Genomics, Chinese Academy of Sciences (GSA: CRA027631) that are publicly accessible at https://ngdc.cncb.ac.cn/gsa (accessed on 18 September 2025).

## Supplementary Materials

The following supporting information can be downloaded at: https://www.mdpi.com/article/10.3390/v17111508/s1, Table S1: Quality-controlled and host-depleted sequence data. Table S2: SARG database annotation results.

## Abbreviations

The following abbreviations are used in this manuscript:

Ppy	Red-billed chough
Cfr	Rook
Cda	Daurian jackdaw
HPAIV	highly pathogenic avian influenza virus
WNV	West Nile virus
NDV	Newcastle disease virus
ARGs	Antibiotic Resistance Genes
PBS	phosphate-buffered saline
vOTUs	viral operational taxonomic units
