# Supplementary material for: Viromic Insights into Gut RNA Virus Diversity Among Three Corvid Species
Source: Viruses. 2025 Nov 17;17(11):1508. doi: 10.3390/v17111508 (PMC12656962; doi:10.3390/v17111508)
Supplement: Supplementary file 1 [file viruses-17-01508-s001.zip › viruses-3918793-supplementary.pdf]

Viromic Insights into Gut RNA Virus Diversity Among Three Corvid Species

Yonggang Dong[1,2], Sitong Fan[2], Lilin Zhu[3], Kirill Sharshov[4,5], Wen Wang[1*]

[1] State Key Laboratory of Plateau Ecology and Agriculture, Qinghai University, Xining 810016, China

[2] College of Eco-Environmental Engineering, Qinghai University, Xining 810016, China

[3] Xining Wildlife Park of Qinghai Province, Xining 810016, China

[4] Federal Research Center of Fundamental and Translational Medicine, Novosibirsk 630117, Russia

[5] Novosibirsk State University, Novosibirsk, 630117, Russia

Corresponding Author

Tel: +86-971-5201533

E-mail: 007cell@163.com (Wen Wang)

Supplementary Materials

TableS1: Quality-controlled and host-depleted sequence data

SampleID	TotalInputReadsPair	host	nohostReadsPair	nohostrate(%)
Ppy1	24521679	17600254	6921427	28.22
Ppy2	30685620	9081145	21604477	70.4
Ppy3	12133131	9256689	2876444	23.7
Ppy4	14065799	10969833	3095968	22.01
Cda1	19085742	14403654	4682090	24.53
Cda2	28637714	24987544	3650172	12.74
Cda3	29093985	25238222	3855765	13.25
Cda4	29950350	6471596	23478756	78.39
Cfr1	32490832	11750301	20740533	63.83
Cfr2	30498598	10654278	19844322	65.06
Cfr3	29068605	10340504	18728103	64.42
Cfr4	28303415	8994086	19309331	68.22

Table S2: SARG database annotation results

GeneID	Identity %	Identity Len	Evalue	DBid	SubType	Type	Mechanism
Cda1__372_1	65	294	2.2e-110	gb|AAC75089.1|ARO:3003577|ugd	ugd	polymyxin	Antibiotic target alteration
Ppy1__1042_1	100	143	1.8e-78	EF468463.1.gene1.p01	TEM-162	beta_lactam	Enzymatic inactivation
Ppy2__130_1	100	170	7.2e-96	gb|APY23677.1|ARO:3001051|TEM-191	TEM-191	beta_lactam	Enzymatic inactivation
Ppy2__1946_1	45.6	114	3.5e-22	gb|AAO47226.2|ARO:3003318| Streptomyces	Streptomyces rishiriensis parY mutant conferring resistance to aminocoumarin	aminocoumarin	mutation
